# Pediatric pulmonary embolism: Unveiling clinical manifestations, diagnostic challenges, and outcomes in Southwest China

**DOI:** 10.1002/pdi3.83

**Published:** 2024-05-23

**Authors:** Dandong Zhao, Qiang Xiong, Ying Lv, Gong Ting, Shuya Lu, Jian Luo, Xiaohong Xie, Mingxiang Zhang, Linli He, Tian Yang, Daiyin Tian

**Affiliations:** ^1^ Department of Respiratory Medicine Children's Hospital of Chongqing Medical University National Clinical Research Center for Child Health and Disorders Ministry of Education Key Laboratory of Child Development and Disorders Chongqing Key Laboratory of Child Rare Diseases in Infection and Immunity Chongqing China; ^2^ Department of Hepatobiliary Surgery Children's Hospital of Chongqing Medical University National Clinical Research Center for Child Health and Disorders Ministry of Education Key Laboratory of Child Development and Disorders Chongqing Key Laboratory of Pediatrics Chongqing China; ^3^ Department of Radiology Children's Hospital of Chongqing Medical University National Clinical Research Center for Child Health and Disorders Ministry of Education Key Laboratory of Child Development and Disorders Chongqing Key Laboratory of Pediatrics Chongqing China; ^4^ School of Nursing Hong Kong Polytechnic University Hong Kong China; ^5^ Yibin Hospital Affiliated to Children's Hospital of Chongqing Medical University Yibin Sichuan China

**Keywords:** children, delayed diagnosis, outcomes, pathology, pulmonary embolism

## Abstract

Pulmonary embolism (PE) leads to obstruction of pulmonary circulation, resulting in increased pulmonary vascular resistance, elevated pulmonary arterial pressure, and increased right heart load. In severe cases, it can lead to cardiac decompensation and life‐threatening conditions. However, clinical studies on PE in children are limited, with many diagnostic and treatment guidelines derived from adult populations. We retrospectively analyzed the clinical manifestations, risk factors, co‐morbidity, and outcomes of PE patients admitted to a large children's hospital in southwest China. A total of 24 children with PE participated, 9 boys (37.5%), aged 0.1–14.6, (median: 8.15 years old). Except for two asymptomatic cases, the duration from symptom onset to the diagnosis of PE varied from 2 to 45 days (median: 12 days). Among these children, 13 (54.2%) patients experienced a delayed diagnosis exceeding 10 days. A total of 7 children died from underlying diseases, and no one met the outcome of recurrent PE or PE‐related death. Among the 17 survivors, complete resolution of PE was in 11 (64.7%) children, partial resolution who progressed to chronic PE was in 3 (17.7%) children, and no follow‐up computed tomography pulmonary angiography was performed in the remaining 3 (17.7%) children. This study revealed that the majority of pediatric PE cases presented with respiratory symptoms, with a considerable proportion initially misdiagnosed as pneumonia, and emphasized the importance of early recognition and appropriate management strategies in improving outcomes for the affected children. Further research is warranted to elucidate the pathophysiology, refine diagnostic algorithms, and establish standardized treatment protocols tailored to the pediatric population.

## Graphical abstract

This retrospective study describes the clinical manifestations, risk factors, co‐morbidity, and outcomes of pulmonary embolism in a large children's hospital in Southwest China. Summary analysis of diagnostic challenges and outcomes on pediatric pulmonary embolism is as follow.

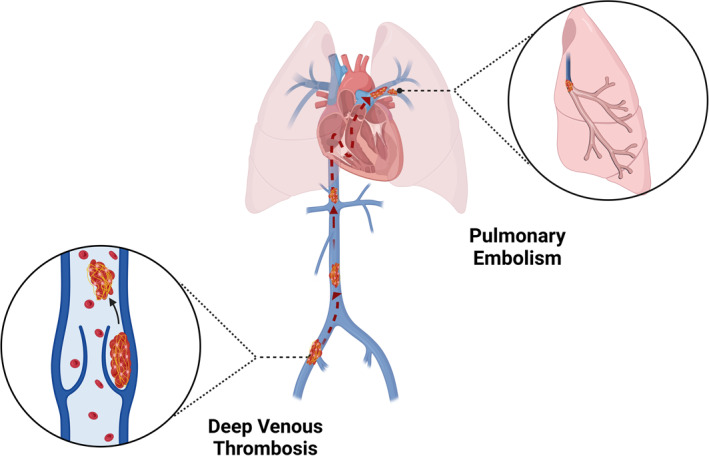

## 1 INTRODUCTION

Pulmonary embolism (PE) is defined as the pulmonary artery and its branches obstructed by an embolus or local thrombus. The most common are thrombus, bacterial thrombus, tumor thrombus, and air embolism. In pediatrics, PE is frequently underdiagnosed due to its nonspecific clinical manifestations, which can be obscured or altered by concurrent diseases, complicating the process of early and accurate diagnosis. Although PE is considerably less common in children than in adults, existing literature indicates that the annual incidence of PE ranges from 0.9 to 5.0 cases per 100,000 children.^1^, ^2^, ^3^ Among hospitalized pediatric patients, the incidence rate ranges from 4.9 to 9.7 per 10,000 children annually.^2^, ^3^, ^4^, ^5^ Despite the lack of statistical significance,^5^ evidence suggests an increasing trend in pediatric PE prevalence. This trend may be attributed to advancements in imaging technology, increased disease recognition, and increased survival of children with chronic diseases.^6^

Pulmonary embolism results in obstruction of pulmonary circulation, leading to increased pulmonary vascular resistance, elevated pulmonary arterial pressure, increased right heart load, and, in severe cases, may cause cardiac decompensation and life‐threatening conditions. However, clinical studies on PE in children are limited. Many prediction models, risk scores, diagnoses, and treatment guidelines for PE in children are derived from adults. Therefore, we aimed to evaluate the clinical features, risk factors, treatment strategies, and follow‐up of pediatric PE cases in our center to improve pediatrician awareness of this rare disease.

## 2 MATERIALS AND METHODS

### 2.1 Study participants

A retrospective study of children with pulmonary embolisms from a large children's medical center in southwest China (Children's Hospital of Chongqing Medical University) was conducted between 1 January 2015 and 31 October 2023. The Research Ethics Board approved this retrospective study and waived informed consent for this study.

Inclusion criteria: ①patients aged 0–18 years; ②PE diagnosed on computed tomography pulmonary angiography (CTPA) or conventional pulmonary angiography or magnetic resonance angiography; ventilation/perfusion (V/Q) scan reporting high probability of PE; Echocardiography demonstrating thrombus in the right ventricle or outflow tract or main pulmonary artery/branch pulmonary arteries or transit; PE was identified on autopsy. Exclusion criteria: the patient had a previous PE.

### 2.2 Procedures and outcomes

According to the American College of Chest Physicians,^7^ high‐risk PE in children has signs or symptoms of shock, cardiopulmonary arrest, or persistent hypotension. Moderate‐risk PE was defined as myocardial necrosis due to elevated cardiac troponin levels, imaging findings of right ventricular strain, or both. Low‐risk PE does not meet the criteria for high or moderate‐risk PE.

The Thrombosis and Hemostasis Scientific and Standardization Subcommittee (ISTH‐SSC) on Pediatric and Neonatal Thrombosis and Hemostasis proposed efficacy and safety outcome definitions as follows.^8^ Efficacy endpoints: all recurrent PE defined as either contiguous progression or new thrombus; PE‐related mortality; and all‐cause mortality. Safety endpoints: Major bleeding (MB) is fatal, with a decrease in Hgb of at least 20 g/L in one 24 h, or in retroperitoneal, pulmonary, or intracranial area, which requires surgical intervention. Minor bleeding: any overt or macroscopic evidence of bleeding.

### 2.3 Data collection and analysis

This retrospective analysis examined the gender, age of onset, additional thrombosis, clinical manifestations, underlying diseases, prothrombotic risk factors, laboratory tests (e.g., D‐dimers, cardiac biomarkers, and thrombophilia test), imaging characteristics, electrocardiogram, therapy, and outcome. Tachycardia and tachypnea were defined according to their age and gender group. If a variable was not documented in the medical records as either present or absent (e.g., recent surgery), it was presumed to be absent.

We used IBM SPSS for statistical analyses. Descriptive statistics were utilized for sample characterization. Potential differences associated with high or moderate‐risk PE versus low‐risk PE were explored in terms of age, gender, clinical presentation (e.g., dyspnea, cough, chest pain, and hemoptysis), D‐dimers levels, chest imaging, and management (thrombolysis, thrombectomy, anticoagulation, mechanical ventilation, or vasoactive agent), as well as outcomes, as detailed. Due to the limited number of cases, associations were assessed using Fisher's exact test, with significance set at *p* < 0.05.

## 3 RESULT

### 3.1 Patient characteristics and basic clinical data

The mean age of the 24 children with PE (9 boys, 37.5%) was 8.15 (0.1–14.6). Except for two asymptomatic pediatric cases, the duration from symptom onset to the diagnosis of PE varied from 2 to 45 days (median: 12 days). Among these cases, 13 (54.2%) experienced a delay exceeding 10 days from symptom onset to the confirmation of PE. Among the clinical characteristics of included patients, 22 (91.7%) had respiratory symptoms, including tachypnea (*n* = 20 [83.3%]), chest pain (*n* = 8 [33.3%]), or hemoptysis (*n* = 6 [25.0%]). More than half had hypoxemia (*n* = 13 [54.2%]), tachycardia (*n* = 17 [70.8%]), and shock or cardiac arrest in 7 (29.2%). We describe the age, gender, combined emolism, risk factors, underlying diseases, risk stratification, therapy, and outcome of the 24 children with PE in Table 1.

**TABLE 1:** Risk factors and outcomes in patients with pulmonary thromboembolism.

Case no.	Age(y)/gender	Type of pulmonary embolism	Combined embolisms	Underlying diseases	Risk factors	Risk stratification	PE location	Therapy	Outcome
1	10y1m/F	Thromboembolism	/	Pneumonia (MP)	Lupus anticoagulant positivity and coagulation dysfunction	Low‐risk	Bilateral, lobar	LMWH + warfarin	Alive with complete resolution
2	7y6m/F	Thromboembolism	Left iliac vein thrombosis	CHD and pneumonia (MP, MRSA)	Coagulation dysfunction, CVC, and glucocorticoids, previous DVT	Low‐risk	Lateral, lobar	LMWH/enoxaparin + warfarin/rivaroxaban	Alive with complete resolution, CTEPH
3	7y11m/F	Thromboembolism	/	CHD, vascular malformation, and pneumonia (MP, MRSA)	Coagulation dysfunction	Low‐risk	Lateral, segmental	LMWH + rivaroxaban	Alive with complete resolution, MB
4	6y2m/F	Thromboembolism	/	Pneumonia (MP)	Coagulation dysfunction	Low‐risk	Lateral, segmental	LMWH	Alive with partial resolution
5	7y11m/M	Thromboembolism	Left atrial thrombosis	Pneumonia (MP)	Lupus anticoagulant positivity and coagulation dysfunction	Low‐risk	Lateral, lobar	LMWH	Alive with complete resolution, MB
6	2m/M	Thromboembolism	/	CHD and pneumonia (MRSA)	/	Low‐risk	Bilateral, left pulmonary artery	LMWH	Alive, no follow‐up
7	13y1m/F	Thromboembolism	Right popliteal vein, femoral vein, and iliac external vein thrombosis	Septic shock (blood culture: MRSA), suppurative hip arthritis, osteomyelitis, and pneumonia	Coagulation dysfunction	High‐risk	Bilateral, segmental	LMWH + rivaroxaban	Alive with partial resolution, MB
8	10y2m/F	Thromboembolism	Right subclavian vein, axillary vein, brachial vein, and superior vein thrombosis	Septicemia (blood culture: S. aureus), pneumonia, osteomyelitis, and cellulitis	Immobilization, recent surgery, and glucocorticoids	Low‐risk	Bilateral, segmental	LMWH + rivaroxaban	Alive, minor bleeding, no follow‐up
9	5m/M	Thromboembolism	/	BPD, CHD, and pneumonia (klebsiella oxytoca)	/	Low‐risk	Lateral, lobar	/	Minor bleeding, death
10	9y7m/M	Thromboembolism	Left atrial thrombosis	AA, pneumonia (fungal infection)	Coagulation dysfunction	Low‐risk	Lateral, right pulmonary artery	/	Death
11	7m/F	Thromboembolism	/	Complex CHD, vascular malformation, and pneumonia (E. coli, SP)	/	High‐risk	Lateral, right pulmonary artery	/	Death
12	13y1m/M	Thromboembolism	Inferior vena cava, iliac vein, and great saphenous vein thrombosis	Pneumonia	Obesity, coagulation dysfunction	High‐risk	Bilateral, main pulmonary trunk	LMWH	Alive, minor bleeding, no follow‐up
13	6y8m/F	Thromboembolism	/	Myocarditis	Coagulation dysfunction	Moderate‐risk	Lateral, segmental	LMWH	Alive with partial resolution, minor bleeding
14	13y6m/M	Thromboembolism	Inferior vena cava, iliac vein, femoral vein, and right popliteal vein thrombosis	Malignancy	Obesity and coagulation dysfunction	High‐risk	Bilateral, left pulmonary artery	Lumbrokinase, LMWH + warfarin/rivaroxaban	Complete resolution, minor bleeding, death
15	8y8m/M	Thromboembolism	Inferior vena cava and internal iliac vein thrombosis	Malignancy, pneumonia, and septicemia	Obesity and coagulation dysfunction	Low‐risk	Bilateral, lobar	Urokinase thrombolytic, LMWH	Partial resolution, minor bleeding, death
16	13y6m/F	Thromboembolism	Right atrial and superior vena vein thrombosis	SLE and pneumonia (SP, MRSA)	Coagulation dysfunction	Low‐risk	Bilateral, lobar	LMWH	Alive with complete resolution
17	8y9m/F	Bacterial embolism	/	Operated CHD (Fallot), vascular malformation, infective endocarditis (blood culture: Gemella haemolysans), and pneumonia	Coagulation dysfunction	Low‐risk	Bilateral, segmental	Thrombectomy	Alive with complete resolution
18	8y/F	Bacterial embolism	/	CHD, infective endocarditis, and pneumonia (Moraxella catarrhalis)	/	Low‐risk	Bilateral, main pulmonary trunk	Thrombectomy	Alive with complete resolution
19	8y10m/F	Bacterial embolism	Left external iliac artery thrombosis and cerebral infarction	CHD and infective endocarditis	Coagulation dysfunction	High‐risk	Lateral, segmental	Thrombectomy, LMWH	Alive with complete resolution, MB
20	12y7m/F	Bacterial embolism	/	CHD and infective endocarditis	Coagulation dysfunction	Low‐risk	Bilateral, main pulmonary trunk	Thrombectomy, LMWH + warfarin	Alive with complete resolution
21	8y6m/F	Tumor embolism	Tumor embolism of superior vena cava	Malignancy and pneumonia	Coagulation dysfunction	Low‐risk	Bilateral, lobar	/	Alive with complete resolution, minor bleeding
22	5y10m/M	Tumor embolism	Tumor embolism of inferior vena cava, right iliac vein, right femoral vein, and left femoral artery	Malignancy, pneumonia (pseudomonas aeruginosa)	Immobilization, recent surgery, and CVC	High‐risk	Bilateral, right pulmonary artery	/	Death
23	2y/M	Tumor embolism	Tumor embolism of inferior vena cava thrombus and left renal vena	Malignancy	Coagulation dysfunction, immobilization, recent surgery, and CVC	High‐risk	Bilateral, main pulmonary trunk	LMWH	Death
24	14y7m/F	Tumor embolism	Tumor embolism of inferior vena cava and left renal vena	Malignancy and urinary tract infection	/	Low‐risk	Bilateral, main pulmonary trunk	/	Alive with complete resolution

Abbreviations: AA, aplastic anemia; BPD, bronchopulmonary dysplasia; CHD, congenital heart disease; CTEPH, chronic thromboembolic pulmonary hypertension; CVC, central venous catheterization; E. coli, Escherichia coli; LMWH, low‐molecular‐weight heparin; MB, major bleeding; MP, mycoplasma pneumonia; MRSA, methicillin‐resistant staphylococcus aureus; PE, pulmonary embolism; S. aureus, staphylococcus aureus; SP, streptococcus pneumoniae.

**FIGURE 1:**
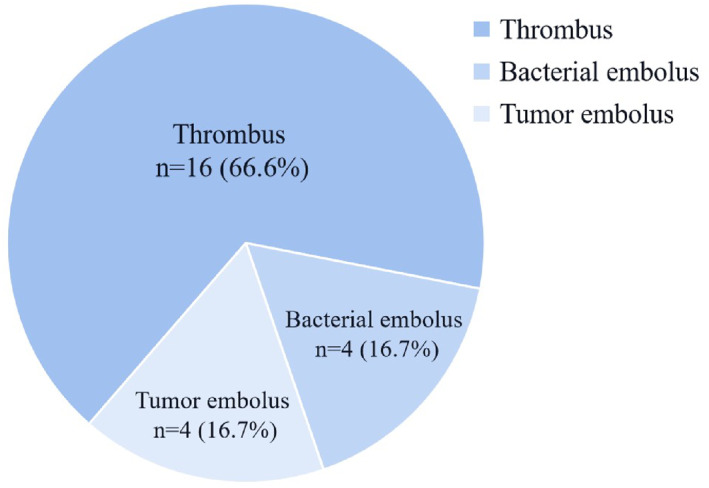
Types of embolism.

Among 24 pulmonary embolisms, 16 (66.6%) were blocked by thromboembolism, 4 (16.7%) were bacterial embolisms and 4 (16.7%) were tumor embolisms (Figure 1).

### 3.2 Underlying diseases and risk factors

At the time of diagnosis, 10 (41.7%) had another venous embolism, including peripheral deep vein thrombosis (*n* = 7), vena cava embolism (*n* = 5), and intracardial thrombus (*n* = 3). A total of 22 (91.7%) had an infection, 9 (37.5%) had congenital heart disease (3 presented with vascular malformation), and 6 (25.0%) had malignant tumor. Among the 22 infected patients, 15 had clear etiological evidence. G(+) bacteria and G(−) bacteria accounted for 53.3% (*n* = 8), respectively. Staphylococcus aureus (*n* = 6 [40.0%]) was the main pathogen, followed by mycoplasma pneumonia (*n* = 5 [33.3%]), and 2 (13.3%) were infected with fungi or streptococcus pneumoniae. In total, 12 (50.0%) pediatric patients underwent immunological evaluations, among whom 2 tested positive for anticardiolipin antibody (aCL), 6 exhibited positive findings on autoimmune antibody profiling, 1 tested positive for rheumatoid factor, and 1 showed positive anti‐neutrophil cytoplasmic antibody. However, in our study, only one child presented with an immune‐related disorder, systemic lupus erythematosus (SLE), a result possibly influenced by the limited sample size in this study.

**FIGURE 2:**
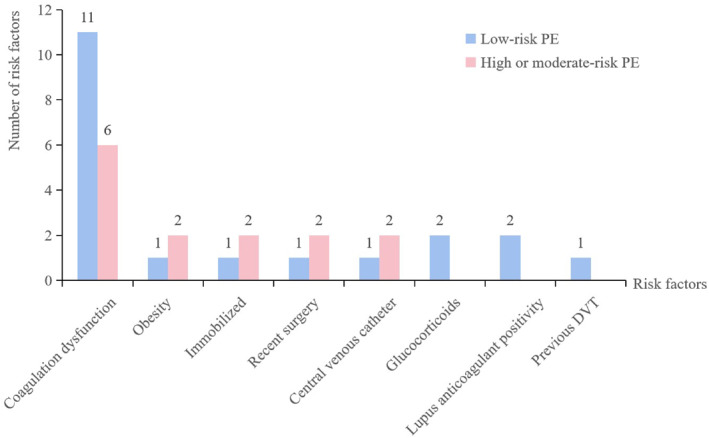
Number of patients with various risk factors. DVT, deep vein thrombosis.

A total of 19 (79.2%) children exhibited at least one risk factor for thrombosis at the time of diagnosis. 17 (70.8%) patients were considered as having coagulation dysfunction, with an elevated D‐dimer (>5 mg/L, *n* = 15) or an elevated FIB (>5 g/L, *n* = 13). Within our cohort, only three children were tested for thrombophilia, with just one testing positive for thrombophilia (factor × deficiency) (Figure 2).

### 3.3 Risk stratification

**FIGURE 3:**
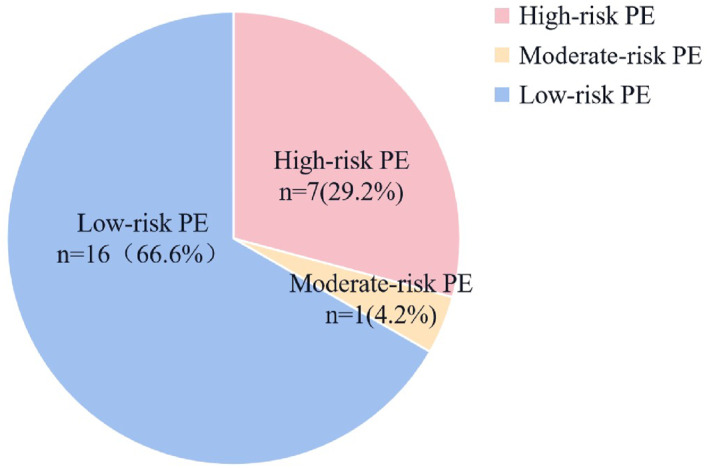
Risk‐stratification.

**FIGURE 4:**
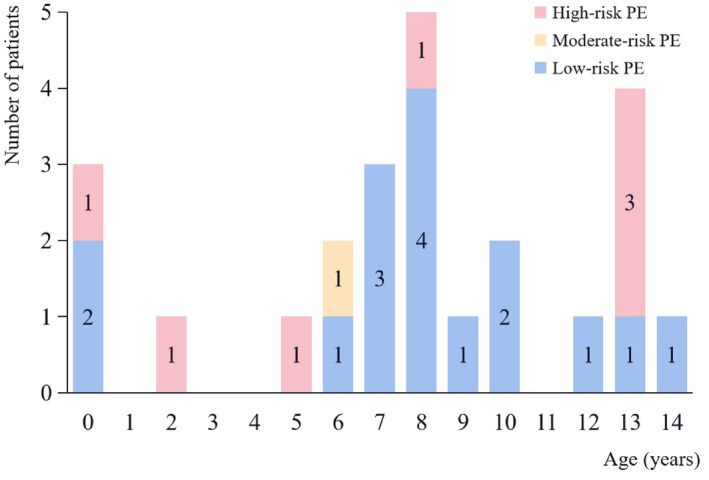
Age distribution of children by PE risk‐stratification.

Ten patients with PE were classified as possible PE by Wells criteria (Wells score > 4), having a sensitivity of 41.6% in our cohort. Six patients with PE met the PE rule‐out criteria (PERC) with and had a false negative rate of 25.0%. The simplified pulmonary embolism severity index (sPESI) classified 21 (87.5%) of the patients with PE as being likely at a high risk of PE (sPESI ≥1). In total, 7 (29.2%) of 24 patients caused by shock or cardiac arrest were classified as high‐risk, and episodes qualified as moderate‐risk were based on the elevation of cardiac biomarkers (*n* = 1 [4%]) (Figure 3). Patients with high or moderate‐risk (*n* = 8 [33.3%]) were younger than patients with low‐risk PE (*n* = 16 [66.6%]) (Figure 4).

### 3.4 Follow‐up

A total of 6 (25.0%) patients received aggressive therapy: mainly systemic thrombolysis (*n* = 2 [8.3%]) or surgical treatment (*n* = 4 [16.7%]). These four cases were treated surgically for confirmed PE caused by bacterial embolus. The majority (*n* = 16 [66.7%]) were treated with low‐molecular‐weight heparin (LMWH), bridging with warfarin (*n* = 4 [16.7%]) and direct oral anticoagulants (DOACs, rivaroxaban, *n* = 5 [20.8%]). In our study, the duration of anticoagulant use varied from 2 weeks to 2 years, reflecting the diverse risk factors and outcomes of patients. A total of 14 (58.3%) patients received other supportive therapies. Comparing the treatment strategies for PE in the low‐risk group (*n* = 16) with the high or moderate‐risk group (*n* = 8), despite the lack of statistical significance in this difference, the latter were more likely to need life support treatment, including those admitted to PICU (7 [43.8%] vs. 6 [75.0%]), ventilator (6 [37.5%] vs. 5 [62.5%]), pulmonary vasodilators (1 [6.3%] vs. 3 [37.5%]), or vasoactive agent (3 [18.8%] vs. 6 [75.0%], *p* < 0.05) (Table 2).

**TABLE 2:** Group comparison according to pulmonary embolism severity.

Number	All (*n* = 24)	Low‐risk PE (*n* = 16)	High or moderate‐risk PE (*n* = 8)	*p*‐value^a^
Male sex	9 (37.5%)	5 (31.3%)	4 (50.0%)	0.412
Age, years (median [IQR])	8.5 (6.33,11.98)	8.5 (7.8,9.8)	8.10 (7.83,8.78)	
Symptoms				
None	2 (8.3%)	1 (6.3%)	1 (12.5%)	1.000
Chest pain	8 (33.3%)	5 (31.3%)	3 (37.5%)	1.000
Hemoptysis	6 (25.0%)	4 (25.0%)	2 (37.5%)	1.000
Tachypnea	20 (83.3%)	12 (75.0%)	8 (100.0%)	0.262
Energielos	15 (62.5%)	7 (43.8%)	8 (100.0%)	0.009
Type of embolism				
Thrombus	16 (66.7%)	11 (68.8%)	5 (62.5%)	1.000
Bacterial embolus	4 (16.7%)	3 (18.8%)	1 (12.5%)	1.000
Tumor embolus	4 (16.7%)	2 (12.5%)	2 (37.5%)	0.578
Risk factors				
Coagulation dysfunction	17 (70.8%)	11 (68.8%)	6 (75.0%)	1.000
Obesity	3 (12.5%)	1 (6.3%)	2 (37.5%)	0.249
Central venous catheter	3 (12.5%)	1 (6.3%)	2 (37.5%)	0.249
Immobilized	3 (12.5%)	1 (6.3%)	2 (37.5%)	0.249
Recent surgery	3 (12.5%)	1 (6.3%)	2 (37.5%)	0.249
Underlying disease				
Infections	22 (91.7%)	16 (100.0%)	6 (75.0%)	0.101
Congenital heart disease	9 (37.5%)	7 (43.8%)	2 (37.5%)	0.657
Malignancy	6 (25.0%)	3 (18.8%)	3 (37.5%)	0.362
PE location				
Bilateral	15 (62.5%)	10 (62.5%)	5 (62.5%)	1.000
Main pulmonary artery	10 (41.6%)	5 (31.3%)	5 (62.5%)	0.204
Lobar	7 (29.2%)	7 (43.8%)	0	0.054
Segmental	7 (29.2%)	4 (25%)	3 (37.5%)	0.647
Therapy				
Ventilator	11 (45.8%)	6 (37.5%)	5 (62.5%)	0.390
Pulmonary vasodilator	4 (16.7%)	1 (6.3%)	3 (37.5%)	0.091
Vasoactive agent	9 (37.5%)	3 (18.8%)	6 (75.0%)	0.021
Surgery	4 (16.7%)	3 (18.8%)	1 (12.5%)	1.000
Thrombolysis	2 (8.3%)	1 (6.3%)	1 (12.5%)	1.000
Anticoagulation	16 (66.7%)	10 (62.5%)	6 (75.0%)	0.667

^a^ Differences compared characteristics of patients with low‐risk PE group and high or moderate‐risk PE group using Fisher's exact test.

**FIGURE 5:**
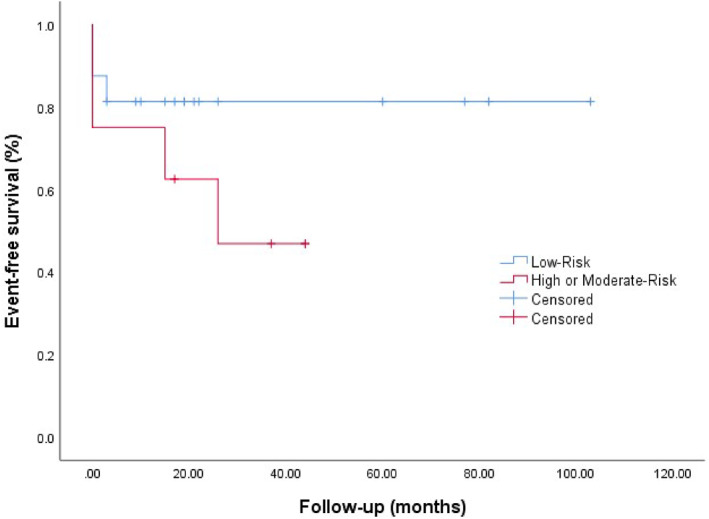
Outcomes per pulmonary embolism severity.

Our follow‐up period ranged from 3 to 105 months. In our cohort, no one met the outcome of recurrent PE or PE‐related death. All‐cause mortality was 3 and 4 in low‐risk PE and high or moderate‐risk PE, respectively, and 4 patients died within 30 days after diagnosis (Figure 5). A total of 4 (16.7%) had MB during the treatment, all of them presented as pulmonary hemorrhages, and 7 (29.2%) showed minor bleeding. However, none of the patients died of hemorrhage. Among the 17 survivors, CTPA showed complete resolution of PE in 11 (64.7%) children, partial resolution in 3 (17.6%), all of them progressed to chronic PE (PE lasted for more than 3 months). All 17 survivors were followed up by chest imaging, 3 had old lesions, 2 had interstitial lung diseases, 2 had bronchiectasis, 1 had obliterans bronchitis, and 1 had bronchiolitis obliterans. Of the 3 patients who underwent pulmonary function testing, 1 was slightly obstructed and 2 had normal ventilatory function. Of the survivors, only 1 showed shortness of breath after exercise, and the rest had no dyspnea. Only 8 children were followed up with cardiac ultrasound, and 2 (25.0%) had pulmonary hypertension.

## 4 DISCUSSION

Pulmonary embolism is characterized by the pulmonary artery and its branches being blocked by emboli or local thrombus. The common emboli are shedding thrombi, tumors, air, bacteria, etc. Tumors can cause coagulation and fibrinolytic system dysfunction, trigger inflammatory cascades, and interact with other complex mechanisms, thereby increasing the susceptibility to massive PE,^9^ which is consistent with our findings. Staphylococcus aureus stands as the most common pathogen associated with PE,^10^ often presenting with high fever, elevated levels of C‐reactive protein, and fibrinogen. Thromboembolism constitutes the predominant manifestation of PE, which can be classified into two types: emboli originating from dislodged deep vein thrombi that travels through the bloodstream and obstructs the pulmonary arterial system, and in situ pulmonary arterial thrombus forms.

Literature indicates a bimodal distribution in the incidence of PE: up to 20.0% of cases constitute a first peak during the neonatal period, and up to 50.0% lead to a second peak during adolescence.^3^, ^11^ The incidence is twice as high among women in the latter group due to estrogen‐related factors such as pregnancy or the use of oral contraceptives. Our cohort did not seem to find a significant bimodal distribution of age at onset, potentially attributed to the absence of oral contraceptives or pregnancy risk factors in our adolescent children. Our discussion will focus on the pathogenesis of pulmonary thromboembolism, the factors contributing to the delayed diagnosis in pediatric populations, and the outcomes of pediatric PE cases.

Virchow Triad^12^ is considered as a crucial factor contributing to thrombus formation, comprising hemodynamic changes, hypercoagulability, and vascular wall endothelial injury. Hemodynamic changes involve slow or stagnant blood flow. Certain chronic diseases necessitate immobilization, leading to stasis or sluggish blood flow within blood vessels, thereby increasing the chances of contact between coagulation factors and platelets with the endothelial cells of vessel wall, consequently elevating the risk of thrombus formation. Hypercoagulability describes a condition characterized by an increased blood‐clotting propensity, typically arising from various factors such as elevated levels of coagulation factors, diminished anticoagulant mechanisms, or compromised fibrinolysis activity. Endothelial injury of the vessel wall, induced by trauma, surgery, inflammation, or chronic vascular diseases, exposes collagen and tissue factors beneath the endothelium, promoting platelet adhesion and aggregation, and initiating the coagulation cascade. The coexistence of any or several of these factors can lead to the formation of thrombosis. For instance, inflammatory diseases such as nephrotic syndrome or SLE, can increase coagulation factor levels, cause endothelial cell damage and dysfunction, platelet activation and aggregation, and prolonged immobilization leading to sluggish blood flow, thereby facilitating thrombus formation. Immunological factors play an important role in the pathogenesis of PE by regulating inflammatory response, thrombosis, and dissolution process. Considering the presence of multi‐site thrombosis in some patients, a comprehensive comprehension of the immunological factors in PE will facilitate a deeper insight into the pathogenesis and progression of the ailment, thereby furnishing novel perspectives and approaches for therapeutic intervention.

Late diagnosis is common in many children with PE, occurring, on average, in 7 days after symptom onset.^13^ In our study, the time between the onset of symptoms and the diagnosis of PE appeared to be longer (range: 2–45 days, median: 12 days). This delay may result from the nonspecific symptomatology of PE, nonverbal patients, and the unreliability of clinical prediction models, including D‐dimer and the PERC, demonstrating poor performance in children.^14^, ^15^

Signs of PE can include dyspnea, cyanosis, hypoxia, tachycardia, arrhythmia, acute right ventricular failure, hypotension, sudden cardiac arrest, or death. Most pediatric patients with PE may not present with typical chest pain, hemoptysis, or dyspnea. Numerous PE cases may exhibit clinical asymptomaticity or have symptoms that are masked by those of the underlying disease,^16^ especially respiratory infections. Many children with PE were misdiagnosed as pneumonia in our study, due to the poor effect of long‐term antibiotic treatment, and the relevant examination was finally improved to confirm PE. When patients on mechanical ventilation suddenly experience unexplained increases in oxygen requirements or persistent dyspnea, vigilance for PE is warranted.^17^ Compared with adults, idiopathic thrombosis is uncommon in children. Instead, most pediatrics with PE are associated with at least one risk factors such as the use of central venous catheters (CVCs), immobility, obesity, sepsis, surgery, trauma, shock, or vascular malformations. In children, the incidence of risk factors is higher compared to the adult population.^18^ Specifically, the most common risk factor for deep venous thrombosis (VTE) in children is the use of a CVC, frequently accompanied by other concurrent risk factors.^11^, ^16^, ^19^ The use of CVCs has significantly enhanced the care of children requiring long‐term venous access. These catheters are widely utilized in children with cancer, sepsis, and chronic diseases, for the administration of intravenous fluids, total parenteral nutrition, and therapies.^20^, ^21^ The D‐dimer test and the PERC are widely used in the initial clinical evaluation of PE in adult patients. Studies indicate that when D‐dimer levels are below 500 ng/mL, the posttest probability of PE is less than 1.9% with low or moderate clinical probability.^22^ A negative D‐dimer and the PERC can safely exclude PE in adult patients. However, the poor performance of clinical predictive models in children with PE is also contributing to the delayed diagnosis. T.T. BISS reported children with PE were equally likely as those without PE to exhibit a D‐dimer level within the normal range.^23^ The PERC demonstrated a sensitivity of 60.0% and a specificity of 46.0% for the diagnosis of PE in children.^24^ In our cohort, the PERC had a false negative of 25.0%. Additionally, the referral of children with PE from other hospitals to our tertiary children's clinic was also a factor leading to delayed diagnosis in our study.

The reported incidence of mortality (all causes) was between 5.5% and 18.0% and the prevalence of recurrence was between 0% and 18.8%.^1^ In our study, no PE‐related death occurred, and all 7 children (29.2%) died of underlying diseases, which may be due to the fact that our research center was a tertiary children's hospital, the patients admitted typically presented with severe illnesses, and a majority of children were not embolized by the main pulmonary artery. Although none of the children in our study had PE recurrence, the recurrence of PE is primarily attributed to factors such as incomplete resolution of previous thrombi, presence of thrombophilia, active malignancy, prolonged immobilization, nephrotic syndrome, and other persistent risk factors. Of the 17 survivors, only 1 showed shortness of breath after exercise, and the remaining patients did not exhibit impaired circulatory or respiratory function. A total of 8 patients had a legacy of chronic lung disease, which was probably not caused by PE but due to underlying diseases such as severe mycoplasma infection. A longer follow‐up period is needed to evaluate the prognosis more clearly and guide the choice of clinical treatment.

## 5 CONCLUSION

This study investigated the clinical manifestations, underlying diseases, risk factors, follow‐up procedures, and reasons for delayed diagnosis among children with PE in a large children's medical center. As the limitation of retrospective study, systematic and standardized intervention in the treatment of children were not feasible, and the differentiation between underlying diseases that were direct causes and those that were mere complications of PE was not adequately discernible. Future research should encompass all kinds of pediatric PE cases to clarify the risk factors, refine clinical prediction models, improve risk stratification, and optimize follow‐up procedures for children.

## AUTHOR CONTRIBUTIONS

Daiyin Tian was involved in designing the review protocol, researching the topic, screening eligible studies, performing data extraction, and revising the article. Dandong Zhao was involved in screening eligible studies, performing data extraction, analyzing the data, making tables, and writing the article. Qiang Xiong and Shuya Lu was responsible for writing‐review and editing. Ying Lv provided the analysis and interpretation of the imaging data. Gong Ting, Jian Luo, Xiaohong Xie, Mingxiang Zhang, Linli He, and Tian Yang interpreted the results and provided expert opinions on the article.

## CONFLICT OF INTEREST STATEMENT

The authors declare no potential conflict of interest.

## ETHICS STATEMENT

The study protocol was approved by the Ethics Committee of the Children's Hospital of Chongqing Medical University (File No. [2024]70).

## Data Availability

The data that support the findings of this study are available from the corresponding author upon reasonable request.
